# Supplementary material for: Medical use of cocaine and perioperative morbidity following sinonasal surgery—A population study
Source: PLoS One. 2020 Jul 30;15(7):e0236356. doi: 10.1371/journal.pone.0236356 (PMC7392254; doi:10.1371/journal.pone.0236356)
Supplement: Attachment — Submitted filename: PONE_cocaine study.pdf [file pone.0236356.s001.pdf]

Medical use of cocaine and perioperative morbidity following sinonasal surgery- a population study

Overall a well written study with a large cohort of patients, with limitations highlighted in the discussion. I only have a few comments and suggestions:

Line 61-62. I thought cocaine's duration of action is 45minutes. Do the authors have any studies to validate the statement that cocaine's analgesic effect can last for hours after anesthetic reversal? (needs an article attached for this sentence)

The study's population limited the subjects to those without previous myocardial infarct/PCI or CABG or congenital heart disease in the last 5 years. Some institutions still use cocaine despite these co-morbidities, albeit in lower doses. It is implied in the study that cocaine should not be used in those with a history of these diseases at all. However, the results of the use of cocaine in this subpopulation, and whether it aggravated the patients' cardiac condition, would be important to know, given cocaine is perceived to be unsafe in patients with the above cardiac conditions. Would the authors comment on this, are there any studies that show cocaine is unsafe in this subpopulation? Is this data available from the database, and could it be analysed separately and included in the study?

What was the cocaine dose used in the patients? Were they all of the same dosage/ solution mixture in every patient or variable depending on surgical preference? Was it a paste or solution or either?

There is no significant difference in primary and secondary outcomes in the cocaine vs non-cocaine group, although higher in cocaine group, which may be explained by the higher number of patients with comorbidities in the cocaine group. In the cocaine group of <=5 pts who had a cardiac event within 48 hours of surgery, how many of them had co-morbidities such as diabetes/hypertension/congestive heart failure, or all 3? Did they have other factors that contributed to their cardiac event with cocaine use? Or is this data not able to be obtained due to risk of patient re-identification? If so, including this in the discussion would be helpful.

What were the other topical anesthetics used by those in the non-cocaine group?

Line 231: should include "in healthy patients or in patients without previous infarct/PCI/CABG" at the end of the sentence, given those with previous MI/ CABG etc were excluded in the study.
