# Supplementary material for: Medical use of cocaine and perioperative morbidity following sinonasal surgery—A population study
Source: PLoS One. 2020 Jul 30;15(7):e0236356. doi: 10.1371/journal.pone.0236356 (PMC7392254; doi:10.1371/journal.pone.0236356)
Supplement: Attachment — Submitted filename: PLOS One Response to Reviewers May 8.20.docx [file pone.0236356.s002.docx]

Editors Comments

Please find below our response to the reviewers in italics.

**1. Please ensure that your manuscript meets PLOS ONE's style requirements, including those for file naming. The PLOS ONE style templates can be found at** [**http://www.plosone.org/attachments/PLOSOne_formatting_sample_main_body.pdf**](http://www.plosone.org/attachments/PLOSOne_formatting_sample_main_body.pdf) **and** [**http://www.plosone.org/attachments/PLOSOne_formatting_sample_title_authors_affiliations.pdf**](http://www.plosone.org/attachments/PLOSOne_formatting_sample_title_authors_affiliations.pdf)

*We have adhered to PLOS ONE’s style requirements.*

**2. In ethics statement in the manuscript and in the online submission form, please provide additional information about the patient records used in your retrospective study. Specifically, please ensure that you have discussed whether all data were fully anonymized before you accessed them and/or whether an IRB or ethics committee waived the requirement for informed consent. If patients provided informed written consent to have data from their medical records used in research, please include this information.**

*Thank you, this information has been included in the methods section.*

**3. We noticed you have some minor occurrence(s) of overlapping text with the following previous publication(s), which needs to be addressed:
doi.org/10.1097/MD.0000000000001106. In your revision ensure you cite all your sources (including your own works), and quote or rephrase any duplicated text outside the Methods section. Further consideration is dependent on these concerns being addressed.**

*The overlapping text has been edited.*

**Reviewer #1: This is a nicely written manuscript on an important topic with a large # of patients across different institutions with opposite practices regarding cocaine use in sinonasal surgery. I generally agree with the conclusions, which do belong in the published literature, but there are some modifications that could make it an even better manuscript.**

**1. Can the authors comment on the impact, if any, of the differences between groups noted in Table 1? The standardized differences for some reached the threshold of relevance.**

*The standardized differences were significant for mean age, rural place of residence, hypertension, surgeon years in practice and volume of surgeries performed per year by the surgeon. We have added the following to the discussion:*

*“We observed baseline differences in the groups which received cocaine versus those that did not receive cocaine. Patients in the cocaine group were more likely to be older, live in a rural location and have hypertension. The province from which were drew our sample is heterogeneous, containing densely populated areas receiving referrals primarily from within cities, as well as less populated smaller cities with referral patterns from primarily rural locations. As we determined our treatment (cocaine) and control (not cocaine) groups by centres that use cocaine versus those that don’t, the differences are likely related to the surrounding geography. Patients who are older are more likely to live in rural locations(24) and to have hypertension.(25)*

*We also observed baseline differences between groups with respect to surgeon years in practice and volume of surgeries per year, with surgeons in the control (not cocaine) group having higher number of years in practice. While surgeons with fewer years in practice usually have less access to operating room time and therefore would perform fewer surgeries per year, the difference found likely reflects the small number of institutions that were represented in our study, with most surgeries performed by a small number of surgeons. Interestingly, and contrary to our findings, two survey studies performed in Canada and in the United States demonstrated that surgeons with fewer years in practice were less likely to use intranasal cocaine.(1,11) Although these survey studies indicate that younger surgeons are less likely to use cocaine, the decision about whether to use cocaine for intranasal surgery is more likely driven by fear of medicolegal concern if an adverse event occurs, the availability of alternate intranasal medications and institutional policies that prohibit intraoperative use of cocaine due to concerns with the storage and dispensing of this controlled substance.”*

**2. Some power calculation is needed, especially since the authors are trying to establish a “negative” conclusion.**

*We did not undertake an* a priori *sample size calculation for two reasons:*

*1) To the best of our knowledge, there is no comparable study from which to derive an expected treatment effect (or harm, in this situation) as most reports have described anecdotal findings and case reports. For calculation of the sample, two potential papers were identified. Qureshi AI et al (2014) is a population-based study that examines the independent association between cocaine use and cardiovascular and all-cause mortality. The second study is the POISE study (2008) which examines the 30-day mortality for non-cardiac surgery comparing placebo versus beta-blockers. Both of these studies have different populations, objectives and follow-up times to our study. Qureshi et al (2014) compared the general population to cocaine drug abusers, the participant cohort age range was 18 to 45 years and the follow-up period was 18 years. The POISE study (2008) included patients undergoing non-cardiac surgery with or at risk of atherosclerotic disease, the mean age of the patients we 69.1 years and the follow-up period of 30 days post-surgery.*

*2) Additionally, as a real-world retrospective cohort analysis (vs. prospective controlled study) a) effect measures from controlled studies would not be appropriate since real-world patients do not meet the strict inclusion/exclusion criteria of controlled trials and b) we are limited by the number of cases that occurred in practice.*

*Experts agree that post-hoc power/sample-size calculations are not recommended when trying to explain a “negative finding” (Walters SJ 2009). Nonetheless, if we were to have applied the effect size from the POISE and Qureshi studies, we would have required 7999 controls (not cocaine) and 3016 exposed (cocaine) (POISE study), or 5862 controls (not cocaine) and 2211 exposed (cocaine) (Qureshi study). Both of these calculations are comparable to the number of patients included in our study (N=7662 controls and N=2887 exposed). Furthermore, unlike other reports which allude to significant harm, we observed a very low event rate (<0.25%) – we further could not account for the differences in the groups because of this low event rate, which would most likely favour the non-cocaine group as they were younger and appeared healthier. Please see below for appropriate references and power calculations.*

*POISE Study Group. Effects of extended-release metoprolol succinate in patients undergoing non-cardiac surgery (POISE trial): a randomised controlled trial. Lancet 2008; 371:1839-47.*

*Qureshi AI, Chaudhry SA, Suri MFK. Cocaine use and the likelihood of cardiovascular and all-cause mortality: data from the Third National Health and Nutrition Examination Survey Mortality Follow-up Study. J Vasc Interv Neurol. 2014; 7(1):76-82.*

*Walters SJ. Consultants’ forum: should post hoc sample size calculations be done? Pharmaceut. Statist. 2009; 8:163-169.*

*PASS was used to calculate the projected sample size for our study with unequal groups. Using Qureshi et al (2014) which examined the risk of mortality in infrequent cocaine users versus cocaine non-user, with 0:0.377 (our study group difference was 2887 cocaine and 7662 control) sample size difference, event rate cocaine non-user 0.0386, event rate cocaine user 0.0466 and age-adjusted RR 1.30 (0.9-1.8), with power of 80 and alpha 0.2. The sample size for cocaine is 2211 and no cocaine 5862 for mortality rate. Follow-up period was ~14 years and patient population was younger than our study.*

**Tests for Two Survival Curves Using Cox's Proportional Hazards Model**

**Numeric Results ────────────────────────────────────────────────────────────**

Hypotheses: H0: HR = 1   vs.   Ha: HR ≠ 1

**Total   Control    Trtmnt                   Actual   Control    Trtmnt**

**Sample   Sample   Sample  Percent    Hazard       Prob       Prob   Control    Trtmnt**

**Size        Size        Size   Control      Ratio      Event      Event    Events    Events**

**Power              N          N1          N2       %N1        HR1       Pev1       Pev2           E1           E2   Alpha     Beta**

0.8000         8073       5862       2211        72.6      1.300      0.039      0.047      226.3      103.0    0.200  0.2000

*Using the POISE study which looked at the 30 day mortality for non-cardiac OR comparing metoprolol group to placebo with 0:0.377 sample size difference, event rate metoprolol group for total 30 day periop mortality 0.0309 and placebo 0.0232 with adjusted HR 1.33 (1.03-1.74), power 80, alpha 0.2. Sample size is 7999 no cocaine group and 3016 cocaine group. This patient group was older than our patient group.*

**Tests for Two Survival Curves Using Cox's Proportional Hazards Model**

**Numeric Results ────────────────────────────────────────────────────────────**

Hypotheses: H0: HR = 1   vs.   Ha: HR ≠ 1

**Total   Control    Trtmnt                   Actual   Control    Trtmnt**

**Sample   Sample   Sample  Percent    Hazard       Prob       Prob   Control    Trtmnt**

**Size        Size        Size   Control      Ratio      Event      Event    Events    Events**

**Power              N          N1          N2       %N1        HR1       Pev1       Pev2           E1           E2   Alpha     Beta**

0.8000       11015       7999       3016        72.6      1.330      0.023      0.031      185.6        93.2    0.200  0.2000

**3. The fisher exact test is not appropriate for large volume data. I believe chi-square is the correct test and do not believe this would change the conclusion. In fact, I suspect the borderline significance of the 30 day findings would be lost (further supporting the authors’ assertions) using the correct but more stringent test.**

*The Chi square test has been added to the table below. Fisher exact test was chosen not based on the number of patients but on the number of expected outcomes. Campbell (2007) focused on seven 2-sided tests (3 versions of the chi-squared test and 4 versions of the Fisher-Irwin test) to calculate maximum Type I errors. The author provides supporting data and concludes “the best policy in the analysis of 2x2 tables from either comparative trials or cross-sectional studies is: 1) Where all expected numbers are at least 1, analyze by the ‘N-1’ chi-squared test (the K. Pearson chi-squared test but with N replaced by N-1); 2) Otherwise, analyze by the Fisher-Irwin test, with two-sided tests carried out by Irwin’s rule (taking tables from either tail as likely, or less, as that observed)”. We believe this provides evidence that the Fisher exact test is the most appropriate test to use for this outcome since many of the expected outcomes are fewer than 1. Reference to this paper has been added to the methods section. Further, upon applying the Chi-squared test as recommended (provided below), SAS returned warnings indicating that the expected cell sizes were too low for accurate estimates.*

*Campbell I. Chi-squared and Fisher-Irwin tests of two-by-two tables with small sample recommendations. Statist. Med 2007; 26:3661-3675.*

| **Table 3. Descriptive statistics of outcomes, by exposure to cocaine during surgery** | | | | | |  | |  |  | |  |  |  |
| --- | --- | --- | --- | --- | --- | --- | --- | --- | --- | --- | --- | --- | --- |
|  | **Unexposed to cocaine (N=7,662)** | | **Exposed to cocaine (N=2,887)** | | **Fisher's** |  | **Chi-square** | | | |  |  |  |
|  | N of events | Event rate (%) | N of events | Event rate (%) | P-value | P-value | P-value | | | P-value |  |  |  |
| Any cardiac event within 48 hours of surgery, N(%) | 0 | 0 | <=5 | <=0.17 | > 0.05 | > 0.05 | < 0.05 | | | < 0.05 |  |  |  |
| Myocardial infarction | 0 | 0 | <=5 | <=0.17 |  |  |  | | |  |  |  |  |
| Coronary artery bypass graft | 0 | 0 | 0 | 0 |  |  |  | | |  |  |  |  |
| Percutaneous coronary intervention | 0 | 0 | <=5 | <=0.17 |  |  |  | | |  |  |  |  |
| Death within 48 hours of surgery, N(%) | 0 | 0 | 0 | 0 |  |  |  | | |  |  |  |  |
| Any cardiac event within 30 days of surgery, N(%) | 0 | 0 | <=5 | <=0.17 | < 0.05 | > 0.05 | < 0.05 | | | < 0.05 |  |  |  |
| Myocardial infarction | 0 | 0 | <=5 | <=0.17 |  |  |  | | |  |  |  |  |
| Coronary artery bypass graft | 0 | 0 | 0 | 0 |  |  |  | | |  |  |  |  |
| Percutaneous coronary intervention | 0 | 0 | <=5 | <=0.17 |  |  |  | | |  |  |  |  |
| Death within 30 days of surgery, N(%) | 6 | 0.08 | <=5 | <=0.17 | > 0.05 |  | > 0.05 | | |  |  |  |  |

**4. The authors state: “Due to potential risk of patient re-identification, institutional policies prohibit the presentation of results of 5 or fewer individuals. Furthermore, exact p-value for the primary outcome could not be presented to avoid the exact number of event rates from being back calculated.” Although knowing the exact number may not change the conclusions at all, It seems IRB approval could allow identification of the individual cases to determine if there were common features that could be identified to account for the event.**

*We consulted the Privacy & Legal Office at ICES and are informed that the data sharing agreements that govern the data we used for analysis explicitly prohibit ICES from reporting cells based on fewer than six observations.  Not even research ethics board approval can overcome this restriction.  The “small cell” rule is designed to minimize the risk that any individual could be re-identified.*

**5. Are there any other questions that can be answered from this large dataset – intraoperative events, bleeding, postop pain?**

*Unfortunately, the dataset does not allow answering any of the questions listed above as they are poorly coded. We were primarily interested in perioperative cardiac events and mortality which are coded well with this dataset.*

**Reviewer #2: Overall an informative study, showing a positive safety profile for cocaine use during sinonasal surgery. This is particularly relevant for those institutions that use cocaine for their procedures. I have suggested the authors consider these points.**

**Page 4 Line 56-57: Specific reference to Ontario, is this relevant to the paper?**

*This sentence has been modified to read “In the United States, approximately 600,000 ambulatory sinonasal surgeries are performed each year” and an appropriate reference has been included.*

**Page 4 Line 61-63: Is there a reference to support the use of cocaine for post operative pain, and additionally the duration of effect.**

*Two references have been added to support the use of cocaine for post operative pain (Tarver et al 1993 and Campbell et al 1992). A study by Foltin et al (2004) demonstrating the duration of plasma levels of intranasal cocaine has also been referenced.*

**Page 8 Paragraph starting Line 144: Are the range of cocaine doses known for the institutions that use cocaine?**

*The dose of cocaine used is 1.4% cocaine in 1:10,000 epinephrine solution, this was confirmed by personal communication with the surgeons at all of the institutions that use cocaine. A statement indicating this was added to the methods.*

**Could the authors summarize the previous largest studies on cocaine use in sinonasal surgery, there seems to be a lack of discussion of the literature. (major revision)**

*The following has been added to the discussion “Our results are consistent with previous large studies in the literature indicating a low rate of cardiac events and mortality following sinonasal surgery. Bhattacharyya (2010) studied perioperative outcomes in over 600,000 patients undergoing sinonasal surgery in the United States over a one year period and found that there were no cases of cardiac arrest.(**2**) The lower rate of cardiac arrest observed in this study may be explained by the use of the National Survey of Ambulatory Surgery database which includes both hospital –based ambulatory surgery and freestanding ambulatory surgery centers.(**2**) All of the institutions included in our study were tertiary care academic hospitals and as a result patients have more comorbidities and the surgeries are more complex requiring in some cases a fellowship-trained Rhinologist.*

*Several trials have been conducted on patients undergoing sinonasal surgery using topical cocaine compared to other agents. The primary outcomes of these studies include pain perception(**4**,* *5**), plasma absorption of intranasal cocaine(**7**,* *26**,* *27**), surgical field visualization and intrapoperative bleeding(**12**,* *28-30**), and adverse events (including cardiovascular changes, electrocardiogram changes, cardiovascular events and mortality)(**2**,* *7**,* *8**,* *28-31**) There was no difference in intraoperative bleeding or surgical field visualization amongst the studies reported in the literature.(**12**,* *28-30**) Further, none of the trials in the literature demonstrate an increased risk of cardiovascular morbidity or mortality association with the use of intranasal cocaine which is in keeping with the results of our study.(**2**,* *7**,* *8**,* *28-31**) The reports of adverse cardiovascular events associated with the use of intranasal cocaine are from case studies.(**9**) Our study along with the trials reported in the literature demonstrate that there is no increased risk of cardiovascular related morbidity or morality associated with the used of cocaine for sinonasal surgery.”*

**Page 13 Paragraph starting Line 224: This paragraph is repetitive and should be restricted.
The authors have postulated that a larger study is not possible. This statement assumes the authors have intimate knowledge of all international databases and their capabilities, which I think is speculative.
Additionally, a statement of “best available data” should instead relate back to why this study is successful given the low event rate, which is the size of the population included.**

*We deleted this paragraph and instead included the following sentence in the second paragraph of the discussion: “In spite of the low event rate we observed, this is currently the best available data demonstrating no difference in cardiac events and mortality in patients that received intranasal cocaine versus those that didn’t given the large sample size of our study.”*

**Page 13 Line 227-228: The sentence starting “We included” is repetitive.**

*This sentence has been deleted.*

**Page 14 Line 242: Is case complexity relevant – could the authors refer to case complexity of already reported cocaine associated morbidity. The previously referenced systematic review showed a range of case complexities at risk.**

*The following sentence has been added to the discussion of limitations “Finally, the complexity of the surgery and the severity of the sinonasal disease is not recorded in the databases that we used, therefore, we could not account for it. Previous studies however, have demonstrated no difference in operative field visualization and intraoperative blood loss with cocaine compared to other topical anesthetics.(**12**,* *28-30**)”*

**Reviewer #3**

**The statistical analysis appears sound, but Im not a statistician so I did not delve into the statistical analysis of the study. Overall this study will appeal to ENT surgeons who still use cocaine for their surgical patients, thus appeal to a narrow range of readers.** **Overall a well written study with a large cohort of patients, with limitations highlighted in the discussion. I only have a few comments and suggestions:**

**Line 61-62. I thought cocaine’s duration of action is 45minutes. Do the authors have any studies to validate the statement that cocaine’s analgesic effect can last for hours after anesthetic reversal? (needs an article attached for this sentence)**

*Several references have been added to support the use of cocaine for post operative pain (Tarver et al 1993 and Campbell et al 1992). A study by Foltin et al (2004) demonstrating the duration of plasma levels of intranasal cocaine has also been referenced. These studies demonstrate that cocaine can peak in plasma concentration from 0-30 after topical nasal administration depending on the formulation used, however, it can still be detected in the plasma for as long as 3 hours* *after intranasal administration. There are no studies that examine the anaesthetic effects of cocaine in the nose longer than 60 minutes after administration.*

**The study’s population limited the subjects to those without previous myocardial infarct/PCI or CABG or congenital heart disease in the last 5 years. Some institutions still use cocaine despite these co-morbidities, albeit in lower doses. It is implied in the study that cocaine should not be used in those with a history of these diseases at all. However, the results of the use of cocaine in this subpopulation, and whether it aggravated the patients’ cardiac condition, would be important to know, given cocaine is perceived to be unsafe in patients with the above cardiac conditions. Would the authors comment on this, are there any studies that show cocaine is unsafe in this subpopulation? Is this data available from the database, and could it be analysed separately and included in the study?**

*The systematic review by Higgins et al (2011) recommends not using intranasal cocaine in patients with a history of cardiovascular disease. The practice of most surgeons who use intranasal cocaine is to avoid the use of cocaine in patients with a history of cardiovascular disease which is why this patient group was excluded. There are no studies that demonstrate that cocaine is unsafe in patients with a history of cardiovascular disease but our intent with this study was to determine the rate of cardiovascular events in a population of patients generalizable to the patients for whom otolaryngologists would use cocaine. The number of patients in our study that were excluded for a previous history of cardiovascular disease or congenital heart disease was 286 patients. Given the low event rate the we observed and the small number of patients with a history of cardiovascular disease or congenital heart disease meaningful results could not be obtained from an analysis of this excluded group of patients.*

**What was the cocaine dose used in the patients? Were they all of the same dosage/ solution mixture in every patient or variable depending on surgical preference? Was it a paste or solution or either?**

*The dose of cocaine used is 1.4% cocaine in 1:10,000 epinephrine solution, this was confirmed by personal communication with the surgeons at all of the institutions that use cocaine. A statement indicating this was added to the methods.*

**There is no significant difference in primary and secondary outcomes in the cocaine vs non- cocaine group, although higher in cocaine group, which may be explained by the higher number of patients with comorbidities in the cocaine group. In the cocaine group of <=5 pts who had a cardiac event within 48 hours of surgery, how many of them had co-morbidities such as diabetes/hypertension/congestive heart failure, or all 3? Did they have other factors that contributed to their cardiac event with cocaine use? Or is this data not able to be obtained due to risk of patient re-identification? If so, including this in the discussion would be helpful.**

*We consulted the Privacy & Legal Office at ICES and are informed that the data sharing agreements that govern the data we used for analysis explicitly prohibit ICES from reporting cells based on fewer than six observations.  Not even research ethics board approval can overcome this restriction.  The “small cell” rule is designed to minimize the risk that any individual could be re-identified.  We an included the following statement in the discussion “We are not able to report details on the patients who experienced outcomes in each group due to the risk of patient re-identification.”*

**What were the other topical anesthetics used by those in the non-cocaine group?**

*The topical anesthetics used in the control (unexposed group) were a combination of 1:10,000 epinephrine, 1:100,000 epinephrine, oxymetazoline 0.05% or lidocaine 1-2%. This was confirmed through personal communication with the surgeons at the control institutions. A statement indicating this was added to the methods.*

**Line 231: should include “in healthy patients or in patients without previous infarct/PCI/CABG” at the end of the sentence, given those with previous MI/ CABG etc were excluded in the study.**

*This paragraph has been deleted at the request of one of the other reviewers.*
