# Supplementary material for: Medical use of cocaine and perioperative morbidity following sinonasal surgery—A population study
Source: PLoS One. 2020 Jul 30;15(7):e0236356. doi: 10.1371/journal.pone.0236356 (PMC7392254; doi:10.1371/journal.pone.0236356)
Supplement: Attachment — Submitted filename: Response to Reviewers June 29.docx [file pone.0236356.s003.docx]

**Response to Reviewers June 29, 2020**

**PONE-D-20-04499R1**

**Medical use of cocaine and perioperative morbidity following sinonasal surgery- A population study**

**PLOS ONEPONE-D-20-04499R1
Medical use of cocaine and perioperative morbidity following sinonasal surgery- A population study**

**Editor Comments:

The responses and revisions with regards to addressing the reviewers comments have been useful. I have a comment and recommend a minor revision. Line 66 to 67 ... lasts for hours after the patient is reversed from anesthesia.... references indicate that a second application of cocaine is needed within an hour of the first application to continue significant effects. Can you please specify how long, instead of simply stating for hours, you would expect the anesthetic effect to last for from the initial application of intranasal cocaine in preparation for endoscopic sinus surgery.**

Thank-you for your comment. On close review of the literature, the duration of anesthetic effect of cocaine is not well defined. We have deleted this sentence.
